# Gut Microbial, Inflammatory and Metabolic Signatures in Older People with Physical Frailty and Sarcopenia: Results from the BIOSPHERE Study

**DOI:** 10.3390/nu12010065

**Published:** 2019-12-26

**Authors:** Anna Picca, Francesca Romana Ponziani, Riccardo Calvani, Federico Marini, Alessandra Biancolillo, Hélio José Coelho-Júnior, Jacopo Gervasoni, Aniello Primiano, Lorenza Putignani, Federica Del Chierico, Sofia Reddel, Antonio Gasbarrini, Francesco Landi, Roberto Bernabei, Emanuele Marzetti

**Affiliations:** 1 Institute of Internal Medicine and Geriatrics, Università Cattolica del Sacro Cuore, 00168 Rome, Italy; anna.picca1@gmail.com (A.P.); jacopo.gervasoni@policlinicogemelli.it (J.G.); aniello.primiano@unicatt.it (A.P.); antonio.gasbarrini@unicatt.it (A.G.); francesco.landi@unicatt.it (F.L.); emanuele.marzetti@policlinicogemelli.it (E.M.); 2 Fondazione Policlinico Universitario “Agostino Gemelli” IRCCS, 00168 Rome, Italy; francesca.ponziani@gmail.com; 3 Department of Chemistry, Sapienza Università di Roma, 00185 Rome, Italy; federico.marini@uniroma1.it (F.M.); alessandra.biancolillo@univaq.it (A.B.); 4 Department of Physical and Chemical Sciences, University of L’Aquila, 67100 Coppito, Italy; 5 Applied Kinesiology Laboratory–LCA, School of Physical Education, University of Campinas, 13.083-851 Campinas-SP, Brazil; coelhojunior@hotmail.com.br; 6 Unit of Parasitology and Unit of Human Microbiome, Bambino Gesù Children’s Hospital IRCCS, 00168 Rome, Italy; lorenza.putignani@opbg.net; 7 Unit of Human Microbiome, Bambino Gesù Children’s Hospital IRCCS, 00168 Rome, Italy; federica.delchierico@opbg.net (F.D.C.); sofia.reddel@opbg.net (S.R.)

**Keywords:** aging, muscle, amino acids, metabolism, systemic inflammation, profiling, biomarkers, multi-marker, physical performance, gut microbiota

## Abstract

Physical frailty and sarcopenia (PF&S) share multisystem derangements, including variations in circulating amino acids and chronic low-grade inflammation. Gut microbiota balances inflammatory responses in several conditions and according to nutritional status. Therefore, an altered gut-muscle crosstalk has been hypothesized in PF&S. We analyzed the gut microbial taxa, systemic inflammation, and metabolic characteristics of older adults with and without PF&S. An innovative multi-marker analytical approach was applied to explore the classification performance of potential biomarkers for PF&S. Thirty-five community dwellers aged 70+, 18 with PF&S, and 17 nonPF&S controls were enrolled. Sequential and Orthogonalized Covariance Selection (SO-CovSel), a multi-platform regression method developed to handle highly correlated variables, was applied. The SO-CovSel model with the best prediction ability using the smallest number of variables was built using seven mediators. The model correctly classified 91.7% participants with PF&S and 87.5% nonPF&S controls. Compared with the latter group, PF&S participants showed higher serum concentrations of aspartic acid, lower circulating levels of concentrations of threonine and macrophage inflammatory protein 1α, increased abundance of *Oscillospira* and *Ruminococcus* microbial taxa, and decreased abundance of Barnesiellaceae and Christensenellaceae. Future investigations are warranted to determine whether these biomediators are involved in PF&S pathophysiology and may, therefore, provide new targets for interventions.

## 1. Introduction

Sarcopenia, the progressive age-related decline in muscle mass and strength/function, is a major determinant of negative health-related outcomes, including disability, loss of independence, institutionalization, and mortality [1,2].

When focusing on the physical domain, sarcopenia shows remarkable clinical overlap with frailty, a geriatric “multidimensional syndrome characterized by decreased reserve and diminished resistance to stressors”, often envisioned as a pre-disability condition [3]. As such, sarcopenia can be considered to be the biological substratum for the development of physical frailty (PF) and the pathophysiologic foundation of adverse PF-related health outcomes [4,5].

Due to the described commonalities, the two conditions have recently been merged into a new entity (i.e., PF & sarcopenia—PF&S) [6] that was operationalized in the context of the “Sarcopenia and Physical fRailty IN older people: multi-componenT Treatment strategies” (SPRINTT) project [7,8].

Multisystem derangements contribute to muscle loss and may ultimately lead to the development of PF&S [9]. Anabolic resistance, chronic low-grade inflammation, and oxidative stress are advocated among the factors contributing to PF&S [10,11]. These mechanisms are enhanced in the setting of physical inactivity and poor nutrition [12,13]. In this scenario, multi-component interventions encompassing physical activity and adapted nutrition are pillars for the prevention of adverse outcomes associated with PF&S [14].

As recently shown by our group, older adults with PF&S are commonly overweight or obese [15], a feature that has been incorporated in the concept of sarcopenic obesity [16]. Compelling evidence indicates that excessive adiposity contributes to physical frailty and functional limitations in advanced age [17,18]. Adipose tissue is metabolically active and promotes systemic inflammation and oxidative stress [19]. In addition, obesity exacerbates fat infiltration within muscles (i.e., myosteatosis), which, in turn, contributes to muscle dysfunction and physical frailty [20,21].

Gut microbiota is a major player in balancing pro- and anti-inflammatory responses in various disease conditions and in relation to nutritional status [22]. Indeed, the existence of a gut-muscle axis has been hypothesized in the context of PF&S [23]. However, the mechanisms whereby changes in gut microbes–host interactions may influence AA availability, systemic inflammation, and muscle homeostasis in PF&S are yet unexplored.

To address this research question, we used data from the “BIOmarkers associated with Sarcopenia and Physical frailty in EldeRly pErsons” (BIOSPHERE) and the Gut-Liver (GuLiver) Axis studies. BIOSPHERE was designed to determine and validate a panel of PF&S biomarkers pertaining to several pathophysiologic domains (i.e., inflammation, oxidative stress, muscle remodeling, neuromuscular junction dysfunction, and AA metabolism) through multivariate statistical modeling [10,11,24]. The GuLiver Axis study was designed to analyze the relationship among gut microbiota, inflammation, and nutritional and metabolic status in people with and without liver disease [25,26].

The availability of these well-characterized cohorts of older adults enabled us to explore the association among gut microbial profiles, systemic inflammation, and metabolic characteristics in PF&S.

## 2. Materials and Methods

### 2.1. Participants

Participants were recruited among enrollees of BIOSPHERE and GuLiver Axis studies. Both studies were approved by the Ethics Committee of the Università Cattolica del Sacro Cuore (Rome, Italy; protocol number BIOSPHERE: 8498/15; protocol number GuLiver Axis: 741). Study procedures and criteria for participant selection have been previously described [24,25].

In both studies, community-dwellers aged 70+ were recruited after signing written informed consent. The presence of PF&S was established according to the operational definition elaborated in the SPRINTT project [7,27]: (a) physical frailty, based on a summary score on the Short Physical Performance Battery (SPPB) [28] between 3 and 9; (b) low appendicular muscle mass (aLM), according to the cutpoints of the Foundation for the National Institutes of Health (FNIH) sarcopenia project [29]; and (c) absence of mobility disability (i.e., ability to complete the 400-m walk test) [30]. The present investigation involved 35 participants, 18 with PF&S, and 17 nonsarcopenic nonfrail (nonPF&S) controls. Gut microbial profiles, circulating inflammatory mediators, and serum AAs and derivatives were assessed.

### 2.2. Measurement of Appendicular Lean Mass by Dual X-ray Absorptiometry (DXA)

aLM was quantified through whole-body DXA scans on a Hologic Discovery A densitometer (Hologic, Inc., Bedford, MA, USA) according to the manufacturer’s procedures. Criteria for low aLM were as follows: (a) aLM to body mass index (BMI) ratio (aLM_BMI_) <0.789 and <0.512 in men and women; or (b) crude aLM <19.75 kg in men and <15.02 kg in women when the aLM/BMI criterion was not met [29].

### 2.3. Blood Sample and Stool Collection

Blood samples were collected in the morning by venipuncture of the median cubital vein after overnight fasting, using commercial collection tubes (BD Vacutainer^®^; Becton, Dickinson and Co., Franklin Lakes, NJ, USA). Serum separation was obtained after 30 min of clotting at room temperature and subsequent centrifugation at 1000× *g* for 15 min at 4 °C. The upper clear fraction (serum) was collected in 0.5 mL aliquots and stored at −80 °C until analysis.

Participants were carefully instructed on the procedures for fecal sample collection. Stool samples were collected at home in a commercial sterile, dry screw-top container. Upon collection, stool samples were delivered to the Human Microbiome Unit at the Bambino Gesù Children’s Hospital (Rome, Italy) and immediately frozen at −80 °C until further processing.

### 2.4. Measurement of Circulating Inflammatory Mediators

A multi-marker immunoassay was used to measure circulating levels of a panel of inflammatory markers [11,25,26,31]. Briefly, a set of 27 pro- and anti-inflammatory mediators, including cytokines, chemokines, and growth factors, were assayed in duplicate in serum samples using the Bio-Plex Pro Human Cytokine 27-plex Assay kit (#M500KCAF0Y, Bio-Rad, Hercules, CA, USA) on a Bio-Plex^®^ System with Luminex xMap Technology (Bio-Rad) (Table 1). Data acquisition was performed with the Bio-Plex Manager Software 6.1 (Bio-Rad) using instrument default settings. Optimization of standard curves across all of the assayed analytes was carried out to remove outliers. Results were obtained as concentrations (pg/mL).

### 2.5. Determination of Circulating Amino Acids

Serum concentrations of 37 AAs and derivatives were determined by ultraperformance liquid chromatography/mass spectrometry (UPLC/MS), as described previously [10]. Briefly, 50 μL of sample was added to 100 μL 10% (w/v) sulfosalicylic acid containing an internal standard mix (50 μM) (Cambridge Isotope Laboratories, Inc., Tewksbury, MA, USA) and subsequently centrifuged at 1000× *g* for 15 min. The supernatant was collected, and 10 μL was mixed with 70 μL of borate buffer and 20 μL of AccQ Tag reagents (Waters Corporation, Milford, MA, USA). The mixture was subsequently heated at 55 °C for 10 min. Samples were eventually loaded onto a CORTECS UPLC C18 column 1.6 μm 2.1 × 150 mm (Waters Corporation) for chromatographic separation (ACQUITY H-Class, Waters Corporation) and eluted at a flow rate of 500 μL/min with a linear gradient (9 min) from 99:1 to 1:99 water 0.1% formic acid/acetonitrile 0.1% formic acid. Analyte detection was performed on an ACQUITY QDa single quadrupole mass spectrometer equipped with electrospray source operating in positive mode (Waters Corporation). AA controls (level 1 and level 2) manufactured by the MCA laboratory of the Queen Beatrix Hospital (Winterswijk, The Netherlands) were used to monitor the analytic process.

### 2.6. Gut Microbiota DNA Extraction, 16S rRNA Amplification, and Sequencing

Total genome DNA was extracted from fecal samples using the QIAmp Fast DNA Stool mini kit (Qiagen, Germany), according to the manufacturer’s instructions.

The V3-V4 region of the 16S rRNA gene (~460 bp) was amplified using the primer pairs 16S_F (5′-TCG TCG GCA GCG TCA GAT GTG TAT AAG AGA CAG CCT ACG GGN GGC WGC AG-3′) and 16S_R (5′-GTC TCG TGG GCT CGG AGA TGT GTA TAA GAG ACA GGA CTA CHV GGG TAT CTA ATC C–3′), reported in the MiSeq rRNA Amplicon Sequencing protocol (Illumina, San Diego, CA, USA). Amplification reactions were set up using a 2× KAPA HiFi HotStart Ready Mix (KAPA Biosystems Inc., Wilmington, MA, USA). AMPure XP beads (Beckman Coulter Inc., Beverly, MA, USA) were employed to clean-up the DNA amplicons. To obtain a unique combination of bar-code sequences, a second amplification step was performed using the Illumina Nextera forward and reverse adaptor-primers (Illumina, San Diego, CA, USA). The final library was quantified after a clean-up step using a Quant-iT™ PicoGreen^®^ dsDNA Assay Kit (Thermo Fisher Scientific, Waltham, MA, USA) and diluted in an equimolar concentration (4 nM).

Samples were sequenced using an Illumina MiSeqTM platform, following the manufacturer’s specifications, generating 300 base-length paired-end reads. Bacterial 16S rRNA amplicon data were analyzed using a combination of the QIIME 1.9.1 software pipeline and the VSEARCH v1.1 pipeline. Fastq-join was used to merge paired-end raw sequences, followed by a split library step (QIIME). After dereplication and chimera checking (VSEARCH), reads were then clustered into operational taxonomic units (OTUs) at 97% identity. Taxonomy of each of 16S rRNA gene sequence was assigned using the UCLUST against the Greengenes 13.8 database (97% sequence similarity).

### 2.7. Statistical Analysis

Analyses were performed using the freely available software environment for statistical computing and graphics R statistics program (version 3.4.0). Sequential and Orthogonalized Covariance Selection (SO-CovSel) statistics were run under Matlab R2015b environment by means of in-house written functions (freely available at www.chem.uniroma1.it/romechemometrics/research/algorithms/).

Descriptive statistics were run on all data. Differences in demographic, anthropometric, clinical, functional characteristics, and inflammatory and metabolic markers between PF&S and nonPF&S participants were assessed via *t*-test statistics and χ^2^ or Fisher exact tests, for continuous and categorical variables, respectively. All tests were two-sided, with statistical significance set at *p* < 0.05.

To compare the gut microbiota alpha diversity between PF&S and nonPF&S participants, Chao1 index was calculated on raw data and differences were assessed by Wilcoxon test.

Data were then preprocessed removing OTUs not seen more than three times in at least 20% of the samples and were normalized using a regularized logarithm transformation (rlog). Differential abundance analysis between PF&S and nonPF&S groups at the phylum, family, and genus levels was carried out using a negative binomial distribution on data normalized by “size factors”, taking into account sequencing depth between samples. Differences in bacterial abundance were reported as log_2_ fold change (log_2_FC). Only comparisons with a log_2_FC > or <±1.5 and an adjusted (Benjamini–Hochberg method) *p* value < 0.05 were considered significant.

After import into MatLab, serum concentrations of inflammatory and metabolic markers and the abundance of gut microbial OTUs were organized into three matrices (Table 2), to be further processed through a multi-block approach. Given its ability to provide accurate predictions and, at the same time, to identify a parsimonious number of relevant variables (putative markers), the analysis was carried out through the recently developed SO-CovSel algorithm [32].

SO-CovSel is a predictive method that couples variable selection (through the CovSel approach) with sequential multiblock modeling, and it can be used to deal with both quantitative and qualitative responses. According to the method, the response(s) to be predicted can be expressed as a linear combination of variables from the different blocks, as described by the following equation:

$$Y=X_{1}B_{1}+X_{2}B_{2}+X_{3}B_{3}$$

The matrices $B_{1},\;B_{2},\;\mathrm{and}\;B_{3}\;$collect the regression coefficients relating the individual blocks to the response(s). Within the multiblock linear regression framework summarized by the previous equation, one of the main peculiarities of the SO-CovSel methods is that not all the variables from the various blocks are used as predictors, but only the most relevant ones, which are selected according to the CovSel algorithm [33]. In CovSel, the first variable is selected as the one having the maximum covariance with the response. The subsequent variables are selected according to the same criterion, but after having orthogonalized both the X and the Y with respect to the contribution of the previously selected predictors, to avoid redundancy. The other main characteristic of the SO-CovSel method, which derives from its analogy with sequential and orthogonalized partial least squares regression (SO-PLS) [34,35], is that the different blocks are sequentially modeled, after having been orthogonalized with respect to the contribution of the previous ones. This avoids scaling issues and allows evaluating whether the block adds new information or it is redundant.

Based on these considerations, for a problem involving three blocks of predictors, as the one addressed in the present study, the SO-CovSel algorithm can be schematically summarized by the following steps:

1. CovSel algorithm is used to select relevant variables and calculate a regression model between the first block and the responses
  $$Y=X_{1sel}B_{1}+E_{1}$$
2. The second block is orthogonalized with respect to the variables selected in the first block
  $$X_{2orth}=\left[I-X_{1sel}\left(X_{1sel}^{T}X_{1sel}\right)X_{1sel}^{T}\right]X_{2}$$
3. CovSel algorithm is used to select relevant variables and calculate a regression model between the orthogonalized second block and the residuals from the first fit
  $$E_{1}=X_{2selorth}B_{2orth}+E_{2}$$
4. The third block is orthogonalized with respect to the variables selected in the first and second blocks
  $$X_{3orth}=\left[I-X_{12sel}\left(X_{12sel}^{T}X_{12sel}\right)X_{12sel}^{T}\right]X_{3}$$
  where
  $$X_{12sel}=\left[X_{1sel}X_{2orthsel}\right]$$
5. CovSel algorithm is used to select relevant variables and calculate a regression model between the orthogonalized third block and the residuals from the second fit
  $$E_{2}=X_{3selorth}B_{3orth}+E_{3}$$
6. An overall prediction model is built as
  $$Y=\hat{Y}+E_{3}=X_{1sel}B_{1}+X_{2selorth}B_{2orth}+X_{3selorth}B_{3orth}+E_{3}$$
  where the predicted response $\hat{Y}$ is calculated as
  $$\hat{Y}=X_{1sel}B_{1}+X_{2selorth}B_{2orth}+X_{3selorth}B_{3orth}$$

The algorithm, described in the steps above for regression (i.e., for the prediction of a quantitative response) can easily be adapted for classification problems, such as the one addressed in the present study. Indeed, by suitably coding the response matrix Y, a classification problem can be straightforwardly turned into a regression one. In particular, for a problem involving two classes, Y is a binary coded vector that takes the value 1 for PF&S participants and 0 for nonPF&S controls. The classification is then accomplished by properly thresholding the value of the predicted response.

## 3. Results

### 3.1. Characteristics of the Study Population

Thirty-five participants were included in the study: 18 older adults with PF&S (mean age 75.5 ± 3.9 years; 56.0% women) and 17 nonPF&S controls (mean age 73.9 ± 3.2 years; 29.0% women). Clinical and demographic characteristics of study participants are presented in Table 3. Age, sex distribution, and number of co-morbid conditions and medications did not differ between groups. Participants with PF&S showed higher BMI than nonPF&S controls.

### 3.2. Features of Gut Microbiota According to the Presence of PF&S

**Figure 1:**
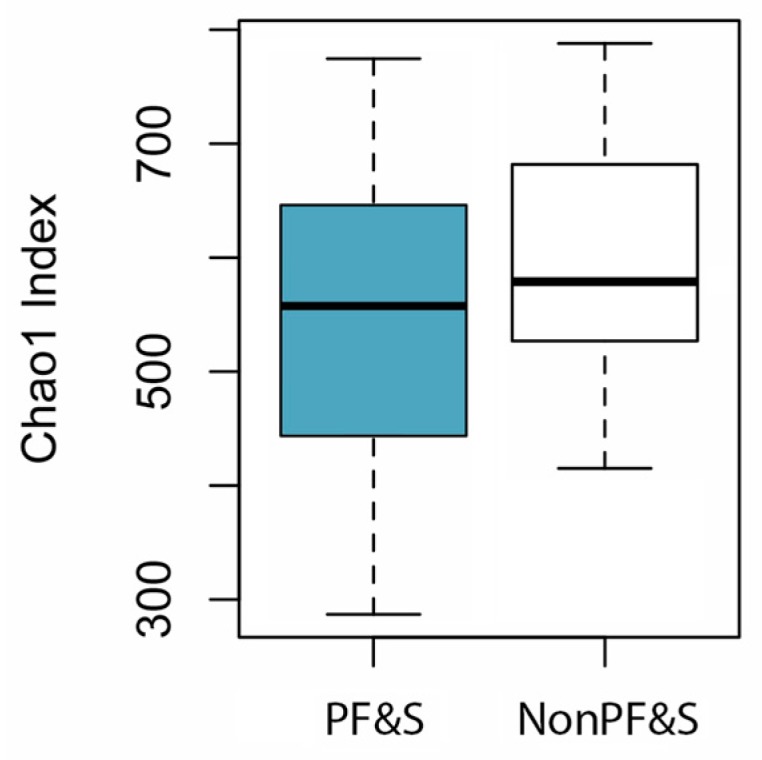
Chao1 index of gut microbial alpha diversity in participants with PF&S and in nonPF&S controls.

No differences in microbial alpha diversity were determined between PF&S and nonPF&S participants (Figure 1).

**Figure 2:**
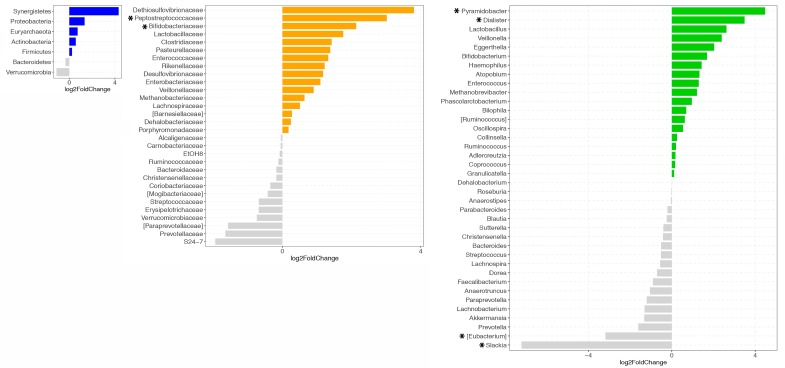
Comparison of gut microbiota relative abundance at the phylum (left panel, blue), family (middle panel, orange), and genus (right panel, green) levels between participants with PF&S and nonPF&S controls. Comparisons with a negative log_2_ fold change (log2FC) are represented in grey. Only comparisons with a log2FC higher or lower than ± 1.5 and an adjusted *p* value < 0.05 are considered significant (*).

The analysis of the differential abundance of microbial taxa between PF&S and nonPF&S groups at the phylum, family, and genus levels showed an increase in Peptostreptococcaceae (*p* = 0.008 Table S1) and Bifidobacteriaceae (*p* = 0.013) at the family level and of *Dialister* (*p* = 0.028), *Pyramidobacter* (*p* = 0.043), and *Eggerthella* (*p* = 0.05) at the genus level, and a depletion in *Slackia* (*p* < 0.0001) and *Eubacterium* (*p* = 0.028) in PF&S participants (Figure 2). No significant differences in the relative abundance of intestinal bacteria phyla were found between groups.

### 3.3. SO-CovSel Analysis

Serum levels of 64 biomolecules, including cytokines, chemokines, growth factors, amino acids, and derivatives, and the abundance of 77 gut microbial taxa, were assayed through multiple analytical platforms. Concentrations of serum mediators are reported in Table S2. Several SO-CovSel models were built using the multimatrix dataset. Among all of the tested models, the one that allowed the best classification of PF&S and nonPF&S participants with the smallest number of variables was selected. This latter was built using only seven analytes (Table 4).

The rate of correct classification was 91.7% for PF&S participants and 87.5% for the nonPF&S group (90.0% in the whole study population), with an average area under the receiver operating characteristic (AUROC) curve very close to 1. When compared with their distributions under the null hypothesis, all of the classification figures of merit were statistically significant (*p* < 0.0001). Among the discriminant analytes selected by the SO-CovSel model, participants with PF&S showed lower levels of MIP-1β. As for the metabolic signature, the level of the aspartic acid was higher in PF&S participants, while that of threonine was higher in the nonPF&S group. Among the gut microbes contributing to the model, *Oscillospira* and *Ruminococcus* were more abundant in PF&S participants, while Barnesiellaceae *and* Christensenellaceae were higher in the nonPF&S group (Table 4).

## 4. Discussion

In this study, we profiled the gut microbiota and determined the levels of inflammatory and metabolic markers in older adults with and without PF&S to investigate whether PF&S is associated with specific profiles of gut microbial taxa and circulating biomolecules. We also applied an innovative multi-marker analytical approach to determine the classification performance of a set of potential biomarkers for PF&S.

Findings from the present study highlight the possibility that changes in gut microbiota composition may be associated with PF&S. Although no differences in microbial alpha diversity were found between PF&S and nonPF&S groups, results from the analysis of the differential abundance of gut microbial taxa showed increased Peptostreptococcaceae and Bifidobacteriaceae at the family level and *Dialister*, *Pyramidobacter,* and *Eggerthella* at the genus level, and depletion of *Slackia* and *Eubacterium* in participants with PF&S. The taxa involved in the association between gut microbiota and PF&S identified in the present study are in-keeping with those previously associated with frailty and biological aging [36,37,38,39,40,41,42].

The application of the SO-CovSel-based analytical strategy by incorporating all of the assayed variables into three different matrices allowed distinguishing participants with PF&S from nonPF&S controls by using only seven markers, among which gut microbes were the most represented (Table 4).

The existence of a gut microbiota profile associated with sarcopenia and involving changes in the abundance of health-related *Bifidobacteria* has previously been shown in rats [43]. While very little is known about a possible functional link between *Oscillospira* and PF&S, the existence of a trait for butyrate-producing bacteria *Ruminococcus* is in-keeping with previously reported associations between *Ruminococcus* abundance and frailty [40]. Indeed, *Oscillospira* represents a more enigmatic and under-studied anaerobic butyrate producer (*Clostridium* clusters IV) often associated with leanness [44], with a metabolism and physiology not fully understood [44,45]. However, the overall apparent dysbiotic shift of gut microbiota towards a greater abundance of butyrate-producing bacteria in PF&S similar to what was observed in higher functioning people may indicate a positive role for these microbes in muscle function. Indeed, butyrate, by reinforcing tight junction assembly and enhancing intestinal barrier function [46], may prevent endotoxin translocation and reduce systemic inflammation [47]. Short-chain fatty acids (SCFAs), including butyrate, also promote fatty acid oxidation, thereby improving muscle bioenergetics [48] and limiting myosteatosis [49,50]. On the other hand, reduced SCFA production may trigger insulin resistance and result in increased fatty acid deposition within the muscle. The ensuing lower muscle quality may further promote insulin resistance, feeding a vicious circle that contributes to the onset and progression of PF&S [51,52]. Whether and how *Ruminococcus* and *Oscillospira* abundance impacts muscle metabolism and function in the context of PF&S warrants further investigation. In this regard, it is noteworthy that the abundance of *Bacteroides* was increased by aerobic training in healthy older women, which was associated with improved cardiorespiratory fitness [53]. Variations in *Ruminococcus* in association with *Eubacterium* and *Eggerthella* were also identified in frail nursing-home residents compared with fit matched community-dwellers [36,37]. This finding was attributed to different dietary patterns and, in particular, to long-term protein supplementation among nursing-home residents [40]. Indeed, diet influences gut microbiota composition and functionality, which may ultimately impact skeletal muscle. While high protein intake has been endorsed as a strategy against sarcopenia [54], protein-enriched diets may shift bacterial metabolism towards AA degradation and fermentation [55]. Hence, the role of gut microbes as transducers of nutrient signaling to the host implies the need of monitoring the composition and function of gut microbiota during nutritional interventions for sarcopenia.

This view is strengthened by the presence of the AAs aspartic acid and threonine among the most relevant mediators in the SO-CovSel model.

Aspartic acid, together with asparagine and glutamic acid is among the AAs providing amino groups and ammonia for the synthesis of glutamine and alanine, whose carbon skeletons can solely be used for the de novo synthesis of Kreb’s cycle intermediates and glutamine [56]. Notably, we previously showed that higher serum levels of aspartic acid, asparagine, and glutamic acid were among the descriptors of the AA signature of older persons with PF&S [10].

Threonine is an essential AA (EAA) that must be provided with the diet to meet nutritional requirements and is relevant for muscle protein turnover and overall metabolism [57]. The finding of threonine as a contributor to the SO-CovSel model in discriminating PF&S participants is in-keeping with a recent work reporting levels of several EAAs, including threonine, as inversely associated with sarcopenia in community-dwelling older adults [58]. Low plasma levels of EAAs were also found in severely frail older people [59]. These findings may be associated to malnutrition (both quantitative and qualitative), a common underlying factor of frailty and sarcopenia [60].

Finally, the relationship observed between the abundance of specific intestinal bacteria, metabolic markers, and serum levels of distinct inflammatory biomolecules suggests the existence of an additional pathway through which changes in gut microbiota may impinge on PF&S pathophysiology. A relationship among gut microbiota composition, chronic inflammation, and age-related conditions was shown in pre-clinical models [61] but not in humans. Furthermore, altered gut microbiota composition has been hypothesized to contribute to anabolic resistance and muscle wasting through promoting chronic inflammation [62,63]. Age-associated alterations in intestinal mucosa permeability (i.e., “leaky gut”) and the resulting systemic absorption of bacterial products may further ignite systemic inflammation [64,65,66]. Although our investigation does not provide mechanistic elements to support such a hypothesis, the relevance of systemic inflammation to PF&S has previously been shown [11]. From this perspective, systemic inflammation would represent one of the effectors of the “gut-muscle axis” that has recently been proposed to contribute to the development of PF&S [23,63,67]. Hence, untangling the relationship among gut microbiota, metabolic changes and muscle homeostasis in advanced age represents a highly promising research area to devise new interventions against PF&S.

Although reporting novel findings, our study presents some limitations that need to be acknowledged. Participants with PF&S had higher BMI than controls, indicative of a sarcopenic obesity phenotype. Because this body composition profile is intrinsic to the PF&S condition [27], the relative contribution of low muscle and excessive adiposity to systemic inflammation, metabolic changes, and gut microbiota composition could not be discerned. The cross-sectional design of the present investigation does not allow inferring causality about changes in gut microbiota and the development of PF&S. Nevertheless, the presence of a gut-muscle axis actively involved in the genesis of frailty and sarcopenia is supported by other studies (reviewed in [67]). Here, for the first time, we show that specific relationships exist among gut microbiota, systemic inflammatory mediators, and metabolic alterations in older adults with PF&S. The relatively small size of the study population comprising only Caucasian people calls for a cautious interpretation of results and impedes generalization of findings to other ethnic groups. Because of the limited sample size, the possible influence of numerous factors, including diet, physical activity, co-morbid conditions, and medications, could not be taken into account in the analysis. Finally, although a fairly large number of metabolic and inflammatory biomolecules were assayed, it cannot be excluded that more robust associations between gut microbiota composition and PF&S might have been obtained through the analysis of a larger range of biomediators.

## Figures and Tables

**Table 1:** List of serum inflammatory biomarkers assayed by multiplex immunoassay.

Cytokines	IFNγ, IL1β, IL1Ra, IL2, IL4, IL5, IL6, IL7, IL8, IL9, IL10, IL12, IL13, IL15, IL17, TNF-α
Chemokines	CCL5, CCL11, IP-10, MCP-1, MIP-1α, MIP-1β
Growth factors	FGF-β, G-CSF, GM-CSF, PDGF-BB

Abbreviations: CCL, C-C motif chemokine ligand; FGF, fibroblast growth factor; G-CSF, granulocyte colony-stimulating factor; GM-CSF, granulocyte macrophage colony-stimulating factor; IFN, interferon; IL, interleukin; IL1Ra, interleukin 1 receptor agonist; IP: interferon-induced protein; MCP-1: monocyte chemoattractant protein 1; MIP: macrophage inflammatory protein; PDGFBB, platelet derived growth factor BB; TNF, tumor necrosis factor.

**Table 2:** Composition of the multi-block dataset used for Sequential and Orthogonalized Covariance Selection (SO-CovSel) analysis.

Data Block	Biological Pathway	Variables
Matrix 1	Inflammation	CCL5, CCL11, IFN-γ, FGF-β, G-CSF, GM-CSF, IL1β, IL1ra, IL2, IL4, IL5, IL6, IL7, IL8, IL9, IL10, IL12, IL13, IL15, IL17, IP-10, MCP-1, MIP-1α, MIP-1β, PDGF-BB, TNF-α
Matrix 2	Protein/amino acid metabolism	1-methylhistidine, 3-methylhistidine, 4-hydroxyproline, α-aminobutyric acid, β-alanine, β-aminobutyric acid, γ-aminobutyric acid, alanine, aminoadipic acid, anserine, arginine, asparagine, aspartic acid, carnosine, citrulline, cystathionine, cystine, ethanolamine, glutamic acid, glycine, histidine, isoleucine, leucine, lysine, methionine, ornithine, phenylalanine, phosphoethanolamine, phosphoserine, proline, sarcosine, serine, taurine, threonine, tryptophan, tyrosine, valine
Matrix 3	Gut microbiota	*Actinobacteria, Adlercreutzia, Aerostipes, Aerotruncus, Akkermansia, Alcaligenaceae, Atopobium, Bacteroidaceae, Bacteroides, Bacteroidetes, Barnesiellaceae, Bifidobacteriaceae, Bifidobacterium, Bilophila, Blautia, Carnobacteriaceae, Christensenella, Christensenellaceae, Clostridiaceae, Collinsella, Coprococcus, Coriobacteriaceae, Cyanobacteria, Dehalobacteriaceae, Dehalobacterium, Desulfovibrionaceae, Dethiosulfovibrionaceae, Dialister, Dorea, Eggerthella, Enterobacteriaceae, Enterococcaceae, Enterococcus, Erysipelotrichaceae, EtOH8, Eubacterium, Euryarchaeota, Faecalibacterium, Firmicutes, Granulicatella, Haemophilus, Lachnobacterium, Lachnospira, Lachnospiraceae, Lactobacillaceae, Lactobacillus, Methanobacteriaceae, Methanobrevibacter, Mogibacteriacea, Oscillospira, Parabacteroides, Paraprevotella, Paraprevotellaceae, Pasteurellaceae, Peptostreptococcaceae, Phascolarctobacterium, Porphyromonadaceae, Prevotella, Prevotellaceae, Proteobacteria, Pyramidobacter, Rikenellaceae, Roseburia, Ruminococcaceae, Ruminococcus, Ruminococcus, S24-7, Slackia, Streptococcaceae, Streptococcus, Sutterella, Synergistetes, Tenericutes, TM7, Veillonella, Veillonellaceae, Verrucomicrobia, Verrucomicrobiaceae*

**Table 3:** Main characteristics of study participants according to the presence of physical frailty & sarcopenia (PF&S).

	PF&S (*n* = 18)	NonPF&S (*n* = 17)	*p*
Age, years (mean ± SD)	75.5 ± 3.9	73.9 ± 3.2	0.2204
Gender (female), n (%)	10 (56)	5 (29)	0.2223
BMI, kg/m^2^ (mean ± SD)	32.14 ± 6.02	26.27 ± 2.55	0.0008
SPPB (mean ± SD)	7.19 ± 1.22	11.24 ± 0.97	<0.0001
aLM, kg (mean ± SD)	17.75 ± 3.17	22.50 ± 2.93	<0.0001
aLM_BMI_ (mean ± SD)	0.55 ± 0.11	0.87 ± 0.15	<0.0001
Number of disease conditions * (mean ± SD)	3.2 ± 1.7	3.0 ± 2.1	0.8046
Number of medications ** (mean ± SD)	3.4 ± 1.2	2.9 ± 1.6	0.1034

Abbreviations: aLM: appendicular lean mass; BMI: body mass index; PF&S: physical frailty & sarcopenia; nonPF&S: nonphysically frail, nonsarcopenic; SD: standard deviation; SPPB: short physical performance battery. * Includes hypertension, coronary artery disease, prior stroke, peripheral vascular disease, diabetes, chronic obstructive pulmonary disease, and osteoarthritis. ** Includes prescription and over-the-counter medications.

**Table 4:** Levels of relevant analytes as resulted from SO-CovSel analysis.

	PF&S (*n* = 18)	nonPF&S (*n* = 17)
MIP-1α (pg/mL)	2.98 (11.04)	10.64 (11.15)
Aspartic acid (µmol/L)	26.95 (9.33)	16.10 (9.28)
Threonine (µmol/L)	109.90 (33.60)	125.80 (55.60)
*Barnesiellaceae* (log2FC)	0.0010 (0.007)	0.0030 (0.003)
*Christensenellaceae* (log2FC)	0.0004 (0.005)	0.0023 (0.004)
*Oscillospira* (log2FC)	0.0147 (0.227)	0.0109 (0.009)
*Ruminococcus* (log2FC)	0.0674 (0.091)	0.0620 (0.058)

Data are shown as median and interquartile range.

## Supplementary Materials

The following are available online at https://www.mdpi.com/2072-6643/12/1/65/s1, Table S1: Differential abundance analysis of bacterial taxa at phylum, family, and genus level in participants with physical frailty and sarcopenia (PF&S) and nonPF&S. Table S2: Serum concentrations of nonsignificant inflammatory mediators, amino acids, and derivatives in participants with and without physical frailty & sarcopenia (PF&S).
