# Supplementary material for: Gut Microbial, Inflammatory and Metabolic Signatures in Older People with Physical Frailty and Sarcopenia: Results from the BIOSPHERE Study
Source: Nutrients. 2019 Dec 26;12(1):65. doi: 10.3390/nu12010065 (PMC7019826; doi:10.3390/nu12010065)
Supplement: Supplementary file 1 [file nutrients-12-00065-s001.zip › Table S2.docx]

**Table S2.** Serum concentrations of inflammatory mediators, amino acid and derivatives in participants with and without physical frailty & sarcopenia (PF&S).

|  | **PF&S (*n* = 18)** | **nonPF&S (*n* = 17)** | **p** |
| --- | --- | --- | --- |
| CCL11 (pg/mL) | 187.75 (211.16) | 169.01 (184.27) | 0.5123 |
| CCL5 (ng/mL) | 8.62 (11.94) | 14.50 (14.23) | 0.6660 |
| IFNγ (pg/mL) | 2.50 (0.42) | 3.22 (2.85) | 0.0043 |
| IL1ß (pg/mL) | 0.93 (0.48) | 1.11 (0.62) | 0.0113 |
| IL1ra (pg/mL) | 149.27 (81.68) | 153.49 (141.39) | 0.3237 |
| IL4 (pg/mL) | 2.21 (2.85) | 3.02 (3.37) | 0.9030 |
| IL6 (pg/mL) | 2.55 (1.01) | 2.81 (0.35) | 0.5713 |
| IL7 (pg/mL) | 4.12 (4.97) | 6.12 (6.55) | 0.3513 |
| IL8 (pg/mL) | 7.70 (7.06) | 13.62 (18.86) | 0.0247 |
| IL9 (pg/mL) | 114.7 (45.42) | 111.23 (28.55) | 0.7130 |
| IL12 (pg/mL) | 2.17 (1.29) | 4.05 (1.84) | 0.3197 |
| IL17 (pg/mL) | 14.91 (7.94) | 18.13 (9.09) | 0.3929 |
| FGF basic (pg/mL) | 39.41 (19.46) | 52.97 (26.63) | 0.4844 |
| GM-CSF (pg/mL) | 1.12 (1.95) | 1.86 (0.67) | 0.2171 |
| IP10 (pg/mL) | 719.64 (836.47) | 614.01 (314.04) | 0.3434 |
| MCP1 (pg/mL) | 29.5 (17.3) | 34.6 (23.5) | 0.0042 |
| MIP-1α (pg/mL) | 2.98 (11.04) | 10.64 (11.15) | 0.2844 |
| MIP-1β (pg/mL) | 155.87 (76.46) | 185.12 (96.54) | 0.7374 |
| PDGF-BB (ng/mL) | 2.49 (1.18) | 4.22 (2.04) | 0.0464 |
| TNF-α (pg/mL) | 35.59 (23.73) | 42.23 (36.60) | 0.3034 |
| α-amino butyric acid (µmol/L) | 19.55 (12.13) | 20.90 (10.20) | 0.3718 |
| β-alanine (µmol/L) | 8.65 (3.50) | 6.40 (4.00) | 0.2750 |
| β-amino butyric acid (µmol/L) | 1.85 (1.83) | 1.20 (1.43) | 0.8534 |
| γ-aminobutyric acid (µmol/L) | 0.40 (0.10) | 0.30 (0.15) | 0.066 |
| 1-methylhistidine (µmol/L) | 4.50 (13.95) | 3.55 (11.20) | 0.6724 |
| 3-methylhistidine (µmol/L) | 4.60 (3.35) | 3.55 (2.90) | 0.4041 |
| 4-hydroxyproline (µmol/L) | 12.90 (9.18) | 18.55 (17.63) | 0.3894 |
| Alanine (µmol/L) | 413.05 (127.00) | 363.50 (138.48) | 0.4877 |
| Aminoadipic acid (µmol/L) | 1.70 (1.53) | 1.35 (1.85) | 0.8763 |
| Arginine (µmol/L) | 112.20 (27.48) | 100.50 (38.25) | 0.9396 |
| Asparagine (µmol/L) | 81.80 (21.30) | 79.60 (23.00) | 0.6125 |
| Aspartic acid (µmol/L) | 26.95 (9.33) | 16.10 (9.28) | 0.0110 |
| Citrulline (µmol/L) | 52.90 (22.05) | 43.00 (15.18) | 0.0968 |
| Cystine (µmol/L) | 30.15 (22.90) | 22.70 (16.28) | 0.2455 |
| Ethanolamine (µmol/L) | 9.45 (2.53) | 8.65 (3.70) | 0.9916 |
| Glycine (µmol/L) | 243.35 (41.65) | 241 (133.85) | 0.5187 |
| Glutamic acid (µmol/L) | 74.59 (19.15) | 65.65 (39.25) | 0.5950 |
| Histidine (µmol/L) | 80.45 (13.95) | 77.00 (13.65) | 0.9502 |
| Isoleucine (µmol/L) | 59.85 (21.05) | 62.85 (32.60) | 0.4324 |
| Leucine (µmol/L) | 130.10 (28.95) | 123.20 (30.28) | 0.5675 |
| Lysine (µmol/L) | 199.90 (31.85) | 209.75 (95.00) | 0.6025 |
| Methionine (µmol/L) | 21.30 (3.63) | 22.75 (8.08) | 0.2469 |
| Ornithine (µmol/L) | 101.50 (39.38) | 115.35 (52.40) | 0.7150 |
| Phenylalanine (µmol/L) | 69.25 (12.93) | 65.65 (18.33) | 0.4521 |
| Phosphoethanolamine (µmol/L) | 0.95 (1.73) | 1.70 (1.48) | 0.7770 |
| Proline (µmol/L) | 199.60 (116.15) | 224.25 (90.43) | 0.5886 |
| Sarcosine (µmol/L) | 1.70 (0.58) | 1.35 (0.85) | 0.0430 |
| Serine (µmol/L) | 125.1 (21.9) | 112.2 (11.4) | 0.0270 |
| Taurine (µmol/L) | 210.10 (39.73) | 215.55 (63.60) | 0.9427 |
| Threonine (µmol/L) | 109.90 (33.60) | 125.80 (55.60) | 0.0720 |
| Tryptophan (µmol/L) | 56.20 (14.43) | 57.70 (25.18) | 0.3495 |
| Tyrosine (µmol/L) | 69.50 (20.63) | 64.85 (44.80) | 0.8196 |
| Valine (µmol/L) | 244.40 (57.13) | 218.90 (59.90) | 0.5808 |

Data are shown as median and interquartile range.

*Abbreviations*: CCL, C-C motif chemokine ligand; FGF: fibroblast growth factor; GM-CSF: granulocyte-macrophage colony-stimulating factor; IFN: interferon; IL: interleukin; IL1Ra, interleukin 1 receptor agonist; IP: Interferon gamma-induced protein; MCP-1: monocyte chemoattractant protein 1; MIP: Macrophage Inflammatory Protein; PDGFBB, platelet derived growth factor BB; PF&S: Physical Frailty and Sarcopenia; TNF, tumor necrosis factor.
