# Supplementary material for: Gut Microbial, Inflammatory and Metabolic Signatures in Older People with Physical Frailty and Sarcopenia: Results from the BIOSPHERE Study
Source: Nutrients. 2019 Dec 26;12(1):65. doi: 10.3390/nu12010065 (PMC7019826; doi:10.3390/nu12010065)
Supplement: Supplementary file 1 [file nutrients-12-00065-s001.zip › Table S1.docx]

**Table S1**. Differential abundance analysis of bacterial taxa at phylum, family and genus level in participants with physical frailty and sarcopenia (PF&S) and nonPF&S controls. Differences are reported as logarithmic fold change (log2FC); positive or negative log2FC indicates higher or lower bacterial abundance compared with the reference group (nonPF&S). Statistical significance is expressed as p value adjusted for multiple comparisons (Benjamini–Hochberg method). Results considered significant (log2FC higher or lower than ±1.5 and adjusted p-value <0.05) are highlighted in bold.

|  |  | PF&S vs. nonPF&S |  |
| --- | --- | --- | --- |
| **Phylum** | **log2FC** | **p value** | **p (adjusted)** |
| *Proteobacteria* | 1.348276229 | 0.024099694 | 0.120498472 |
| *Synergistetes* | 4.327660626 | 0.016706822 | 0.120498472 |
| TM7 | 0.80979191 | 0.488496484 | 0.550305632 |
| *Actinobacteria* | 0.573970184 | 0.325538921 | 0.550305632 |
| *Firmicutes* | 0.240352339 | 0.495275069 | 0.550305632 |
| *Verrucomicrobia* | -1.11148872 | 0.250701658 | 0.550305632 |
| *Cyanobacteria* | 0.823547699 | 0.244186703 | 0.550305632 |
| *Bacteroidetes* | -0.335354132 | 0.459100567 | 0.550305632 |

|  |  | PF&S vs. nonPF&S |  |
| --- | --- | --- | --- |
| **Family** | **log2FC** | **p value** | **p (adjusted)** |
| ***Peptostreptococcaceae*** | **3.017514365** | **0.00024552** | **0.00859319** |
| ***Bifidobacteriaceae*** | **2.135639009** | **0.00075421** | **0.013198676** |
| *Clostridiaceae* | 1.422420187 | 0.02846486 | 0.249067524 |
| *Dethiosulfovibrionaceae* | 3.803955233 | 0.022881276 | 0.249067524 |
| *Lachnospiraceae* | 0.508566205 | 0.130671333 | 0.436744995 |
| *[Paraprevotellaceae]* | -1.572650114 | 0.210802877 | 0.436744995 |
| *Prevotellaceae* | -1.649303855 | 0.127566154 | 0.436744995 |
| *Lactobacillaceae* | 1.758378333 | 0.140228965 | 0.436744995 |
| *Veillonellaceae* | 0.905849201 | 0.223484194 | 0.436744995 |
| Other | 0.864429462 | 0.223796921 | 0.436744995 |
| *Rikenellaceae* | 1.224875183 | 0.064261368 | 0.436744995 |
| *Erysipelotrichaceae* | -0.682161667 | 0.224611712 | 0.436744995 |
| Other | -2.032170649 | 0.152191051 | 0.436744995 |
| *Enterococcaceae* | 1.324931514 | 0.208849549 | 0.436744995 |
| Other | -0.762360918 | 0.143323144 | 0.436744995 |
| *Enterobacteriaceae* | 1.100733468 | 0.126441438 | 0.436744995 |
| *Desulfovibrionaceae* | 1.177882339 | 0.173488945 | 0.436744995 |
| *Pasteurellaceae* | 1.38235537 | 0.13741247 | 0.436744995 |
| S24-7 | -1.939194607 | 0.325611725 | 0.569820519 |
| *Streptococcaceae* | -0.678633083 | 0.316566848 | 0.569820519 |
| *[Mogibacteriaceae]* | -0.422044038 | 0.407768056 | 0.679613427 |
| Other | 0.719663068 | 0.505372398 | 0.707521357 |
| *Verrucomicrobiaceae* | -0.737569037 | 0.449737294 | 0.707521357 |
| Other | -0.701746276 | 0.492092147 | 0.707521357 |
| *Methanobacteriaceae* | 0.640188 | 0.491135331 | 0.707521357 |
| *Coriobacteriaceae* | -0.346855138 | 0.536359345 | 0.722022195 |
| *Ruminococcaceae* | -0.117294284 | 0.751348209 | 0.876572911 |
| *Bacteroidaceae* | -0.175245958 | 0.698724285 | 0.876572911 |
| *Dehalobacteriaceae* | 0.24568757 | 0.740614945 | 0.876572911 |
| *[Barnesiellaceae]* | 0.277686003 | 0.742487496 | 0.876572911 |
| *Porphyromonadaceae* | 0.178838793 | 0.797090959 | 0.899941405 |
| *Christensenellaceae* | -0.176975694 | 0.831401155 | 0.909345014 |
| *Carnobacteriaceae* | -0.051501661 | 0.952554321 | 0.970949598 |
| *Alcaligenaceae* | -0.046637408 | 0.970949598 | 0.970949598 |
| EtOH8 | -0.077409104 | 0.952094457 | 0.970949598 |

|  |  | PF&S vs. nonPF&S |  |
| --- | --- | --- | --- |
| **Genus** | **log2FC** | **p value** | **p (adjusted)** |
| ***Slackia*** | **-7.201348166** | **1.29E-07** | **7.20E-06** |
| **Other** | **3.108067774** | **0.000184875** | **0.005176488** |
| ***Dialister*** | **3.488523694** | **0.001959649** | **0.02830451** |
| ***[Eubacterium]*** | **-3.183327561** | **0.002021751** | **0.02830451** |
| ***Pyramidobacter*** | **4.466986361** | **0.003856958** | **0.043197929** |
| ***Bifidobacterium*** | **1.684978922** | **0.005869584** | **0.050329016** |
| ***Eggerthella*** | **2.028560191** | **0.006291127** | **0.050329016** |
| *Lactobacillus* | 2.619183831 | 0.023908338 | 0.167358364 |
| *Veillonella* | 2.392217783 | 0.028281189 | 0.17597184 |
| Other | 1.422450556 | 0.051359751 | 0.287614608 |
| *Dorea* | -0.716504218 | 0.076412641 | 0.389009807 |
| *Prevotella* | -1.604055323 | 0.136435281 | 0.459462311 |
| *Faecalibacterium* | -0.906476913 | 0.108586221 | 0.459462311 |
| Other | 0.884588087 | 0.13947963 | 0.459462311 |
| *Atopobium* | 1.326035529 | 0.130336394 | 0.459462311 |
| Other | -0.714550677 | 0.128143688 | 0.459462311 |
| *Haemophilus* | 1.431284091 | 0.113604216 | 0.459462311 |
| *Akkermansia* | -1.317551745 | 0.188668664 | 0.494591567 |
| Other | 0.855625457 | 0.215967095 | 0.494591567 |
| *Enterococcus* | 1.294161643 | 0.209254327 | 0.494591567 |
| Other | -0.832490321 | 0.159568408 | 0.494591567 |
| *Oscillospira* | 0.536046887 | 0.187034504 | 0.494591567 |
| Other | -1.267910907 | 0.220799807 | 0.494591567 |
| *Methanobrevibacter* | 1.207451843 | 0.200064206 | 0.494591567 |
| *Lachnobacterium* | -1.306069409 | 0.198135817 | 0.494591567 |
| Other | 1.180780616 | 0.285998178 | 0.500579098 |
| Other | -2.374372331 | 0.235621334 | 0.500579098 |
| Other | -1.539383038 | 0.283086255 | 0.500579098 |
| *[Ruminococcus]* | 0.6232365 | 0.286045199 | 0.500579098 |
| Other | 0.647672459 | 0.252089196 | 0.500579098 |
| Other | -0.460898971 | 0.248859824 | 0.500579098 |
| *Anaerotruncus* | -1.042880722 | 0.261765396 | 0.500579098 |
| *Paraprevotella* | -1.200747937 | 0.357021489 | 0.588035393 |
| *Bacteroides* | -0.512617456 | 0.351764662 | 0.588035393 |
| *Lachnospira* | -0.560581432 | 0.397519688 | 0.6360315 |
| *Bilophila* | 0.691257326 | 0.410917005 | 0.639204229 |
| *Streptococcus* | -0.52585956 | 0.432388547 | 0.65442591 |
| *Phascolarctobacterium* | 0.960293275 | 0.454286401 | 0.669474697 |
| Other | -0.459873921 | 0.578702037 | 0.830956771 |
| *Blautia* | -0.245879756 | 0.605299388 | 0.847419143 |
| Other | -0.156109989 | 0.681857427 | 0.867818544 |
| *Collinsella* | 0.251545057 | 0.667370768 | 0.867818544 |
| *Christensenella* | -0.422233608 | 0.661958532 | 0.867818544 |
| *Ruminococcus* | 0.200678758 | 0.662544355 | 0.867818544 |
| *Parabacteroides* | -0.209600636 | 0.755691789 | 0.881640421 |
| *Sutterella* | -0.404711334 | 0.74567097 | 0.881640421 |
| *Coprococcus* | 0.161664863 | 0.712006155 | 0.881640421 |
| Other | -0.416100006 | 0.737392142 | 0.881640421 |
| *Adlercreutzia* | 0.175791699 | 0.822435412 | 0.937342302 |
| Other | -0.174779587 | 0.83691277 | 0.937342302 |
| Other | -0.096068981 | 0.874332355 | 0.94158869 |
| Other | -0.09861827 | 0.860753892 | 0.94158869 |
| *Granulicatella* | 0.109713827 | 0.896053746 | 0.946773769 |
| *Anaerostipes* | -0.045959871 | 0.94348252 | 0.976472693 |
| *Roseburia* | -0.023672553 | 0.95903568 | 0.976472693 |
| *Dehalobacterium* | 3.98E-05 | 0.999955388 | 0.999955388 |
